# Conceptualising workplace loneliness: a qualitative investigation with UK workers

**DOI:** 10.1007/s00127-025-02925-0

**Published:** 2025-05-20

**Authors:** Bridget T. Bryan, Elena Triantafillopoulou, Vaughan Parsons, Louise Arseneault, Timothy Matthews

**Affiliations:** 1 Social, Genetic and Developmental Psychiatry Centre, Institute of Psychiatry, Psychology and Neuroscience, King’s College London, London, UK; 2 Occupational Health Service, Guy’s and St Thomas’ NHS Foundation Trust, London, UK; 3 School of Life Sciences and Medicine, King’s College London, London, UK; 4 School of Human Sciences, University of Greenwich, London, UK

**Keywords:** Employment conditions, Loneliness, Psychosocial workplace exposure, Qualitative research, Work

## Abstract

**Purpose:**

Loneliness has been identified as an important risk factor for mental health problems, and concern about its impact on workers’ health and wellbeing has grown in recent years. While a body of workplace loneliness research is emerging, the degree to which existing definitions of the phenomenon reflect workers’ experiences has not been investigated. This study aims to develop an evidence-based conceptualisation of workplace loneliness that can inform future research and interventions aiming to improve workers’ mental health and wellbeing.

**Methods:**

Semi-structured interviews exploring experiences of social connection, loneliness and work were conducted with a diverse sample of 31 UK-based workers. The interviews were supplemented with a social mapping task. Interview data were analysed inductively using reflexive thematic analysis. Social maps were analysed using a thematic analysis approach informed by a visual semiology framework.

**Results:**

Three themes were identified, in which loneliness was conceptualised as disconnection from (1) colleagues, (2) one’s organisation, and (3) society. Across each theme, disconnection and loneliness were experienced as an unfulfilled desire to feel that one’s authentic self was understood, valued or belonged as a result of one’s work or occupation.

**Conclusions:**

Workplace loneliness comprises not only dissatisfaction with interpersonal relationships at work, but also a sense of disconnection from larger social groups and structures, particularly one’s employing organisation and society as a whole. Definitions of workplace loneliness that acknowledge the role of the social and organisational context, as well as professional relationships, are needed to better reflect the lived experience of loneliness at work.

**Supplementary Information:**

The online version contains supplementary material available at 10.1007/s00127-025-02925-0.

## Introduction

Unprecedented changes to working patterns during the COVID-19 pandemic and increased awareness of the negative impact of loneliness on health [1] have raised concerns about loneliness among workers and its implications for health and productivity [2]. Loneliness is an important risk factor for mental and physical health problems including depression, anxiety [1], cardiovascular disease [3] and diabetes [4], as well as poor socioeconomic outcomes and difficulties in the job market [5, 6]. A growing body of research linking loneliness experienced in the workplace with burnout, low job satisfaction, reduced job performance [7] and turnover [8] points to work-related loneliness as a threat to workers’ health, wellbeing and career prospects, and as a cost to employers and the economy more broadly. Within this context, workplace loneliness has become a priority for governments and employers, with the UK All Party Parliamentary Group on Tackling Loneliness calling on employers to address loneliness within their organisations [2], and leading loneliness charities offering advice on how employers can tackle loneliness among their staff [9, 10].

Loneliness is typically defined as subjective dissatisfaction with the quality or quantity of one’s relationships [11]. Workplace loneliness is most broadly understood as loneliness that is experienced specifically within the workplace or resulting from aspects of an individual’s job or work [12]. In the emerging literature on work-related loneliness, the common definition of loneliness as dissatisfaction with interpersonal relationships has often been transposed onto the workplace, with workplace loneliness most often defined as dissatisfaction with professional relationships [7, 13]. However, no studies have explored workers’ lived experiences of loneliness and the degree to which this definition maps onto the workplace has not been examined. In light of the subjective nature of loneliness [11], it is essential that conceptualisations of workplace loneliness are rooted in workers’ lived experiences.

Further, while existing definitions focus on loneliness within dyadic collegial relationships, such conceptualisations have been critiqued for failing to consider the role of wider social structures and processes in individual experiences of loneliness [14]. Recent sociological research indicates that a sense of non-belonging within societal norms can be an important aspect of loneliness [15, 16], while longer standing psychological research has highlighted that connection to wider social groups, such as a local community, also plays a role [17]. These broader, collective sources of connection may be particularly relevant in the workplace, as work offers individuals membership to their occupational group and facilitates the development of a social identity through the fulfilment of social norms [18]. However, these theoretical developments have rarely been applied in workplace loneliness research, such that important aspects of work-related loneliness may not be captured using existing definitions or measurement tools.

A conceptualisation of workplace loneliness that reflects the experiences and perspectives of workers is needed to answer calls from governments, employers and the public to better understand and address loneliness in the workplace. This study aims to develop a conceptualisation of workplace loneliness rooted in the lived experiences of workers by using qualitative methods to investigate how they describe, experience and make sense of loneliness at work both before and during the COVID-19 pandemic.

## Data and methods

### Participants and recruitment

A purposive maximum variation sampling strategy [19] was used to recruit a diverse sample of workers with varied experiences of loneliness and work. Individuals aged 20 or older who had been in work in the UK at some time since March 2020 were eligible. Participants were recruited using Instagram and Facebook advertisements over September to November 2021 (full strategy in Supplement A). Prospective participants who clicked on an advertisement could express interest in participating by recording their contact details on the study website. Individuals who expressed interest were contacted with the information sheet and consent form (Supplement B). Participants received a £20 voucher as a thank you for participating. This project was approved by the King’s College London Research Ethics Office (HR/DP-20/21-22104).

The sample consisted of 31 participants working in a range of industries and with varied experiences of work during the COVID-19 pandemic (Table 1). Participants were diverse in gender, ethnicity and disability status. They were aged between 21 and 59 years, with the majority aged under 40 years. Nine participants (29%) reported feeling lonely some of the time, often or always, mirroring prevalence in the UK population [20, 21]. Fourteen participants (45%) scored above the threshold for likely minor psychiatric disorder, suggesting a degree of elevated distress compared to the general population of UK employed adults [22].

### Data collection

#### Pre-interview survey

A short survey was administered to facilitate description of the sample and add context to the interviews [23]. The survey was administered using Qualtrics [24] before each interview and gathered demographic, loneliness and psychological distress data. The interviewer did not access survey responses aside from participants’ occupation before the interview.

Loneliness was measured using the 3-item University of California Los Angeles (UCLA) loneliness scale [25] and one direct question about how often the participant felt lonely. Psychological distress was assessed using the 12-item General Health Questionnaire (GHQ-12; [26]) (full survey in Supplement C). The GHQ-12 was scored 0-0-1-1 with a score of four or higher indicating likely minor psychiatric disorder [27].

#### Semi-structured interviews

In-depth accounts of participants’ experiences of work, loneliness and social connection were collected in semi-structured interviews. Prepared questions explored participants’ occupational histories, workplace relationships and feelings of connection and loneliness (full interview guide in Supplement D). While the advertising strategy introduced the study in the context of the COVID-19 pandemic (Supplement A), the interviews focused on experiences both before and during the pandemic. The interviews were conducted online using Zoom between October 2021 and January 2022 and lasted between 51 and 84 min.

**Table 1:** Sample demographic characteristics and occupational background

Characteristic	*N*	%
**Gender**		
Woman	20	65%
Man	10	32%
Non-binary/other gender	1	3%
**Age**		
21–29 years	4	13%
30–39 years	20	65%
40–49 years	2	6%
50–59 years	5	16%
**Race and ethnicity**		
Arab or Arab British	1	3%
Asian ethnicities	5	16%
Black ethnicities	7	23%
White ethnicities	18	58%
**Disabled**,** D/deaf or long-term health condition**		
Yes	5	16%
No	23	74%
Prefer not to say	3	10%
**Industry type**		
Accommodation, food services or tourism	2	6%
Administrative and support services	3	10%
Construction or electricity, gas, steam and air conditioning supply	2	6%
Education	7	23%
Human health & social work activities	5	16%
Professional, scientific & technical activities or financial & insurance activities	5	16%
Public administration, defence and social security	1	3%
Wholesale and retail trade, repair of motor vehicles	3	10%
Other activities or industries	3	9%
**COVID-19 pandemic working patterns**		
Worked at usual workplace	17	55%
Worked from home	26	84%
Reduced work hours	5	16%
Was furloughed	3	10%
Worked as an essential worker	6	19%
Lost job or stopped working	6	19%
Was unable to work for more than two weeks because of sickness or disability	4	13%
**Loneliness**	***Mean***	***SD***
UCLA-3 score (range = 3–9)	4.74	1.65
**Psychological distress**		
GHQ-12 score (range = 0–12)	3.87	4.01

*N* = 31

#### Social mapping task

A social mapping task supplemented the interviews. Mapping methods, in which participants draw a ‘map’ of their social network [28], can capture aspects of social life difficult to access in interviews, including the complexity of social networks and the meaning participants place on relationships [29]. As such, the mapping task enhanced collection of data on participants’ relationships and workplace social environments.

The interviewer initiated the task around 20 min into the interview. She sent participants a link to the online drawing tool AutoDraw before demonstrating how to use the tool. Participants were then asked to open the tool and draw a map of their relationships at work. Participants could take as long as they needed to create the drawing; this ranged from 4 to 16 min. After completing their drawing, participants shared their screen to show their map, and the interview resumed. All participants were asked to describe the meaning of their drawing. Subsequent discussion of loneliness and social connection at work also made explicit reference to participants’ drawings through follow up questions in most interviews. Drawings were saved for analysis.

Twenty-nine participants created a drawing. One participant had difficulty using the tool and instructed the interviewer in creating a drawing on her behalf. Two participants did not create a drawing because of time limitations.

### Data analysis

Interview and drawing data were analysed inductively using reflexive thematic analysis [30]. Interview transcripts were analysed in NVivo 14 [31]. The first author led the analysis, first immersing herself in the data. She then coded the data before grouping related codes into initial themes, which were developed and refined through discussion with co-authors. The second author revisited 12 transcripts to consider how well the thematic structure ‘fit’ the data. The first and second authors discussed any discrepancies. These discussions added richness to the analysis and informed the final theme structure.

The drawings were analysed using a thematic analysis approach informed by a visual semiology framework [32] that supported analysis of the visual content of the maps in conjunction with participants’ explanations of their drawing. Visual semiology asserts that, like language, the use and meaning of visual elements and symbols will display regularities which can be made the subject of relatively formal description [32]. As such, visual semiology offers a framework for interpreting the meaning of visual elements, such as colour choice, symbols and composition that we used to interpret the maps in hand with participants’ verbal explanations. During the coding phase, participants’ social maps were considered alongside the transcripts and coded for analytically salient visual features. After a preliminary thematic structure was developed, the maps were revisited to identify patterns across the dataset and were associated with related themes where appropriate.

### Researcher positioning and reflexive practice

The first author led the study and conducted data collection. She is a white Australian woman, was a PhD student during the study and has experience working in academia and the service sector in the UK and Australia. The other authors are white researchers employed in UK universities. They have varied migration histories and experiences of work both within and outside academia.

We engaged in reflexive practice using two main strategies, reflexive journals [33] kept by the first and second authors, and formal and informal reflective discussions during analysis and write up (full detail in Supplement E).

## Results

**Fig. 1:**
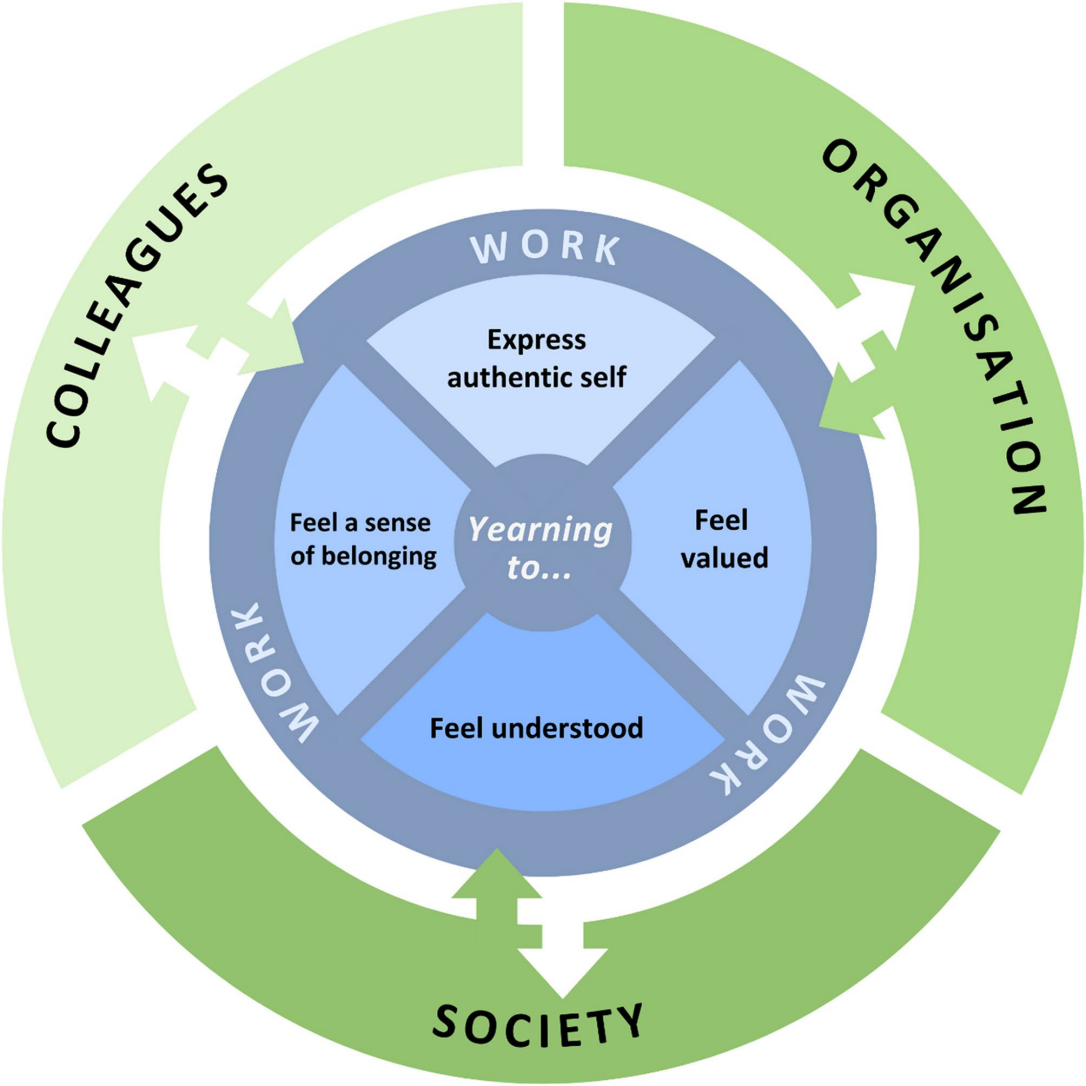
Visual representation of thematic structure

Participants’ accounts of workplace loneliness clustered into three themes, in which loneliness was conceptualised as disconnection from one’s (1) colleagues, (2) organisation, and (3) society (Fig. 1). No single participant reported experiencing loneliness related to all three entities; many participants reported disconnection from one while expressing satisfaction with their connection to another. Across each theme, loneliness was experienced as a yearning to feel a sense of belonging, or to feel that one’s authentic self is valued or understood at, or as a result of, their work.

### Theme 1: disconnection from colleagues

Workplace loneliness was often experienced as dissatisfaction with the quantity or quality of one’s professional connections. Feeling connected to colleagues was particularly important, with relationships with clients, customers and students rarely mentioned as sources of loneliness. This was evident in Jack’s account, in which his loneliness centres on having few interactions with other teachers:

*Yesterday I didn’t interact with any adult and that’s lonely… It feels a bit like lone working at times*,* with students*,* obviously.*

Jack, teacher, aged 30–39.

This account mirrors that of other participants who had limited opportunities to interact with colleagues. For these workers, even frequent interactions with clients, students or customers did not satisfy their need for social interaction at work, leaving them feeling “empty” and “alone.”

In addition to dissatisfaction with the quantity of interactions with colleagues, workplace loneliness was typified by dissatisfaction with the quality of these connections. Loneliness was often

The outer layer (green) represents the three themes and social entities that participants experienced workplace loneliness in relation to: their colleagues, organisation and society. The inner layer (blue) illustrates the aspects of loneliness that cut across the three themes, particularly the yearning to feel that one’s authentic self is understood, valued or belongs.

**Fig. 2:**
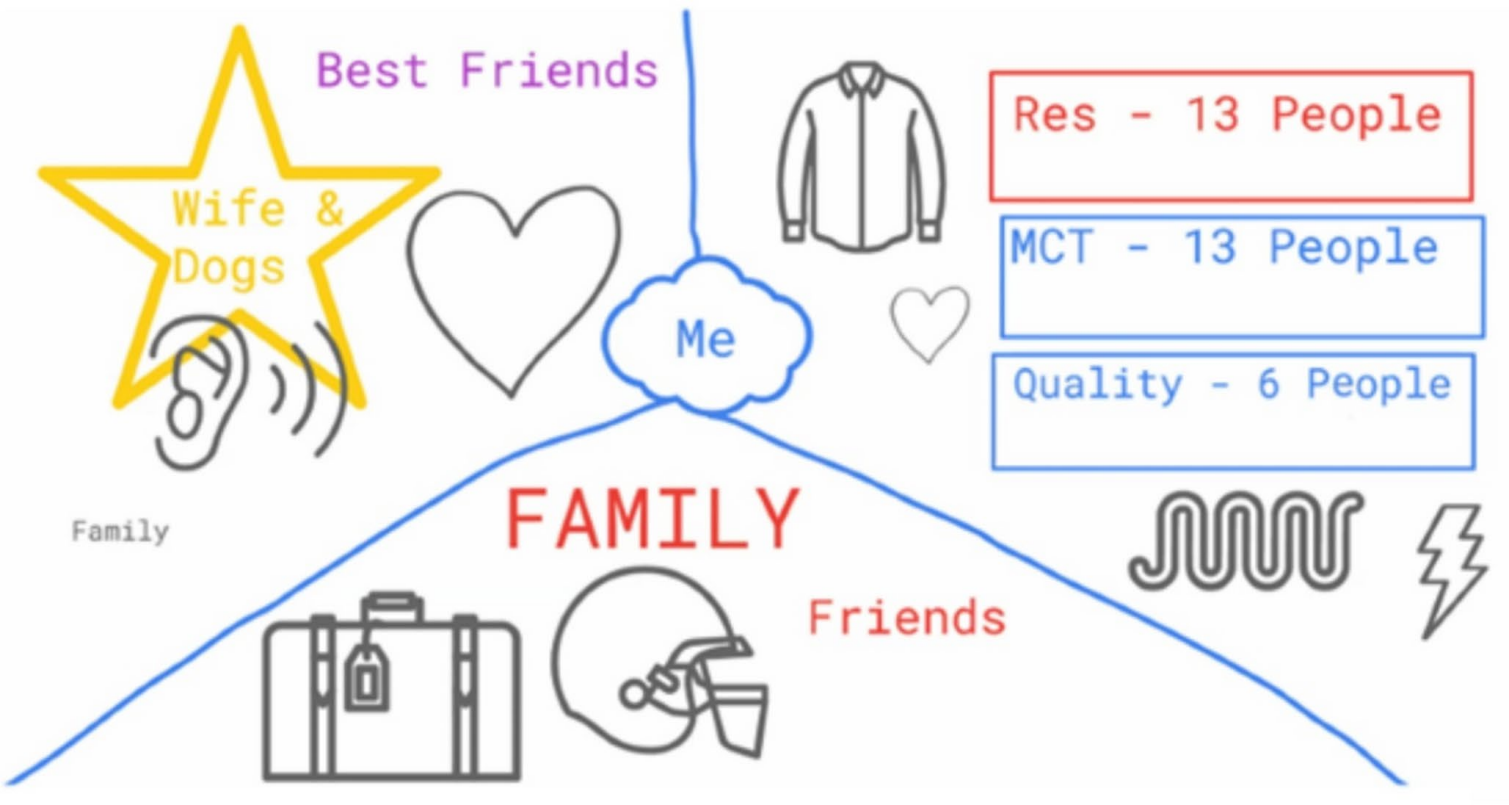
Damian’s social map

Characterised by longing for supportive collegial relationships, with many participants emphasising the importance of workplace friendships and pointing to times in which they lacked intimate connection at work as essentially lonely. The importance of meaningful workplace relationships is illustrated in Damian’s drawing, in which professional relationships take up a third of his social life and are positioned above his relationships with family and friends (Fig. 2). For Damian, work was a source of meaningful relationships:

Excerpt from Damian’s description of his drawing: “*The other [top right-hand] side*,* for me*,* it’s kind of this work side. So*,* it always starts off quite formal for me*,* I always think, at work. And then a little bit of love*,* and a little bit of care always creeps in and you can’t really shake it off… But it’s almost about making connections. I think that*,* that environment here [work segment] is probably where I make more of my friends. I’m not someone really for going out to the pub or meeting new people and going to clubs or social events to meet people*,* I normally always make really strong connections through work. And then they move either into this pocket at the bottom [representing family and friends outside of work] or they move into this pocket [points to bottom section representing his wife and best friends] depending on how we value each other’s time.”*

*The other [top right-hand] side, for me, it’s kind of this work side. So, it always starts off quite formal for me*,* I always think*,* at work. And then a little bit of love, and a little bit of care always creeps in*,* and you can’t really shake it off.*

Damian, operations manager, aged 30–39.

These meaningful relationships were ones in which workers felt they were understood and could express their authentic self candidly. This was articulated by Noah:

*I’ve definitely felt lonely at work in the past in my previous roles where*,* like I mentioned*,* I didn’t have those people*,* those work friends that I can be myself with.*

Noah, marketing manager, aged 30–39.

In her recount of workplace loneliness, Melissa echoes this desire to express herself authentically and shows how feeling she was not understood by her colleagues constituted loneliness:

*Loneliness*,* in my previous jobs I felt that because it was an in-person environment but I just didn’t really have any connection to any of the staff… You can get along with different types of people and that’s fine*,* but if you don’t really feel that connection*,* I suppose I’m quite a sarcastic person*,* and quite a blunt person. So*,* when I feel like I have to filter myself around people I don’t really feel that comfortable or that welcomed.*

Melissa, employment advisor, aged 30–39 years.

In highlighting needing to “filter” herself, Melissa centres the unfulfilled desire to speak authentically in her construction of workplace loneliness. Many participants echoed this sentiment, describing the desire to “be” themselves and the sense that they had to “put on different hats” or “wear a mask” as central to their loneliness at work. James’ definition of loneliness similarly centred on concealing one’s authentic feelings:

*I think loneliness for me would be defined as - I think it is a lot broader than saying someone who is on their own… I would define it as just somebody that doesn’t have anyone to talk to*,* or that they might be afraid to talk to someone about it. They’d rather keep things hidden and they’re probably worried about the consequences of opening up.*

James, construction worker, aged 30–39 years.

This sense of needing to “filter” oneself was particularly common among minoritised participants, such as participants of colour and LGBTQIA + participants. These workers described feeling that they and their experiences were not understood by their colleagues, forcing them to present a sanitised version of themselves or maintain emotional distance from others in order to “fit in.”

In addition to a desire for meaningful relationships, most participants also described loneliness as a yearning for less emotionally intimate connection at work. This was most often described in the context of remote working, where loneliness was regularly defined by wishing to be physically near others, or as missing momentary, casual interactions with colleagues. This was particularly true for Lydia, who described loneliness while working remotely as “I missed my colleagues, I just missed office life.” For Lydia, returning to the office and working near colleagues relieved her loneliness, despite working mostly independently:

*It’s better now… This is weird*,* because I actually like to sit far away from them [colleagues] and I still need peace to get on with my work*,* but I like them to be there.*

Lydia, financial services professional, aged 30–39 years.

**Fig. 3:**
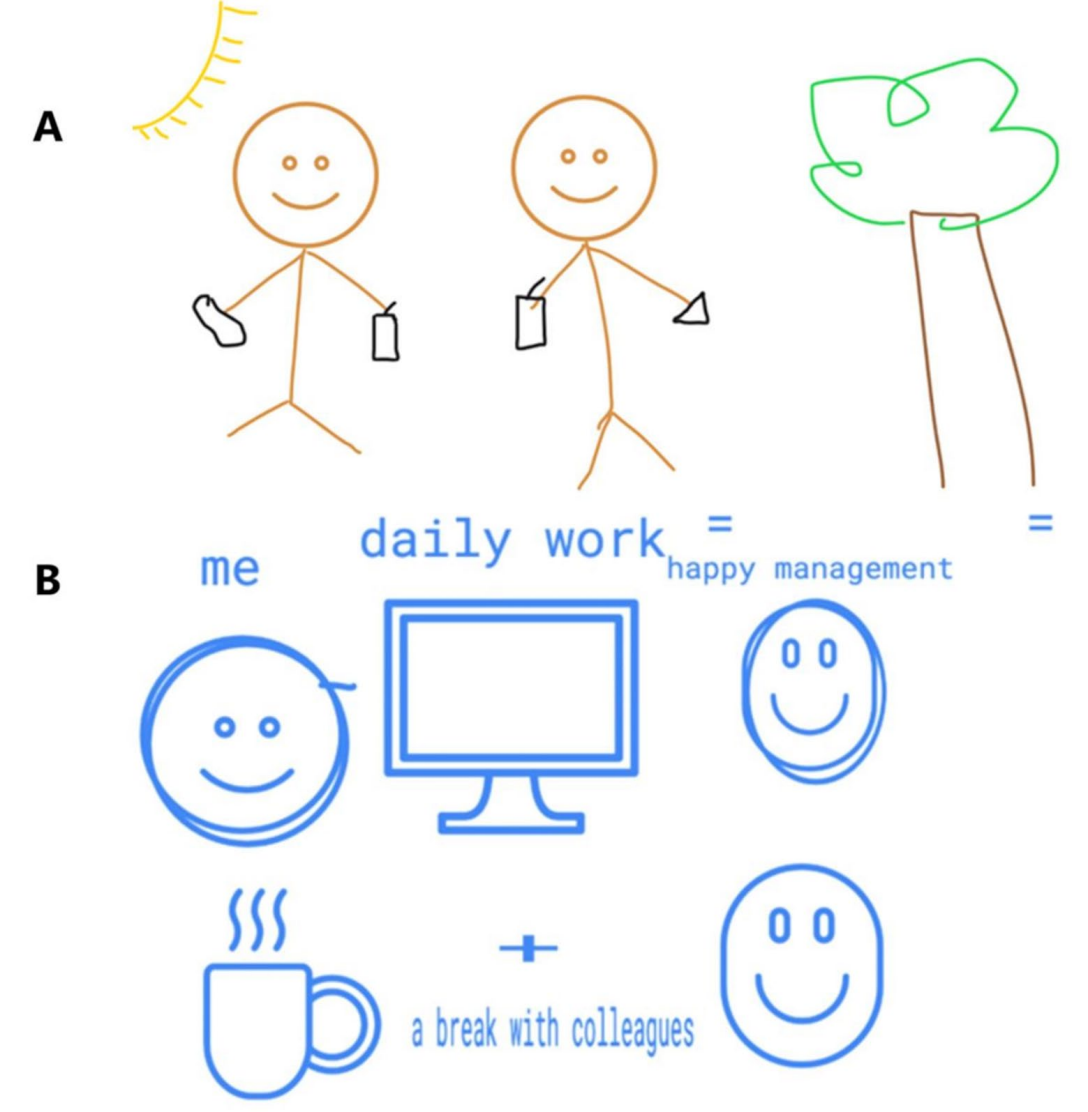
Amal (**A**) and Tegan’s (**B**) social maps

This desire for commonplace interactions with colleagues is illustrated in Amal and Tegan’s drawings (Fig. 3) in which they depicted their preferred working relationships as sharing a meal or cup of tea with a colleague. When asked who they had depicted themselves with, both Amal and Tegan stated that, rather than any specific person, they had drawn themselves with “just any” colleague. In doing so, both emphasised their desire for “simple,” mundane interactions rather than intimate connection in their descriptions of workplace loneliness.

### Theme 2: disconnection from the organisation

In addition to dissatisfaction with relationships with colleagues, many participants described workplace loneliness as feeling disconnected from the organisation that they worked for. For these participants, not feeling “part” of their organisation constituted loneliness, over and above their relationships with other individuals at work. As in relationships with colleagues, these experiences of loneliness were underscored by unfulfilled desires to feel valued and understood, encapsulated in Kim’s explanation of why workers may feel lonely:

*A lot of people maybe work in jobs where they just feel like a number and they don’t feel like a valued individual member of the company.*

Kim, marketing executive, aged 21–29.

Damian’s definition of loneliness similarly centred on feelings of belonging within his organisation, despite frequent interactions with colleagues:

*For me*,* loneliness is kind of that sense of feeling that you don’t belong*,* or that you’re not part of the larger group… I would 100% say that work has a, has an issue with being able to create or to stop loneliness… Particularly in my most recent role at [company]*,* we’d be on these calls all day*,* every day*,* but it felt really lonely. I don’t know anything about the business*,* I don’t know anything about policy or process*,* nothing like that*,* and yet*,* I’ve got all these people around me. So yeah*,* I absolutely agree that the workplace has a real bearing on whether or not you can or can’t be lonely.*

Damian, operations manager, aged 30–39.

In these descriptions of loneliness, the organisation was constructed as a social entity in and of itself that was greater than the sum of its individual members such that the organisation could be a source of connection aside from any individual within it. Organisation-wide policies and communication materials were described as expressing the attitudes or will of an organisation and as indicating whether an employee was valued, supported or accepted by their organisation. This was evident in Naomi’s longing for support during a period of poor mental health:

Naomi: As much as you can have those colleagues around you, you can feel, you can feel really lonely and just like, just like you don’t fit into the company. I’ve certainly felt like that when I first started, especially when I was struggling a bit more with my mental health.

Interviewer: Can you tell me a bit more about when you were feeling lonely? Especially if you had those colleagues around you?

Naomi: I just think there genuinely isn’t much support for mental health within my organisation, which has been, I have been disappointed in it… Organisations can do a lot more behind mental health awareness. Obviously, sharing [phone] numbers for Samaritans and people that you can talk to during your downtime, or having somebody within an organisation that’s dedicated, or even a portal, where you can find information that’s helpful if you’re struggling.

Naomi, salesperson, aged 21–29.

In naming leave policies, online portals and awareness raising activities, Naomi emphasised institutional sources of support for people experiencing mental health difficulties, instead of support from other individuals at work. In doing so, she voices a yearning to feel cared for and valued by her employer and locates her feelings of loneliness within her relationship with the organisation, rather than her interpersonal relationships within it.

**Fig. 4:**
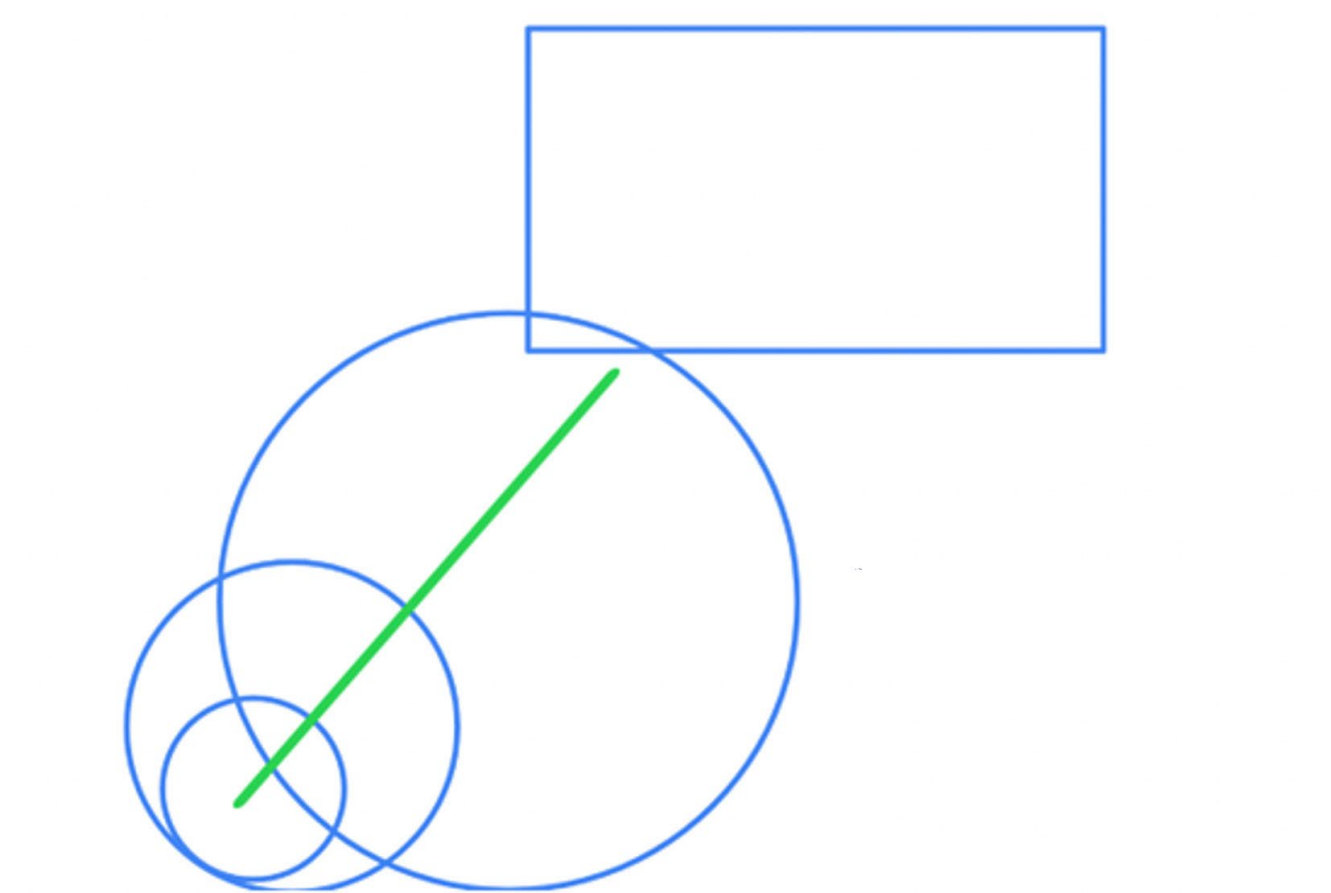
Melissa’s social map

These feelings of estrangement from one’s organisation were often distinct from workers’ satisfaction with their interpersonal relationships at work. This distinction is illustrated in Melissa’s map of her relationships at work (Fig. 4). In her drawing, the circles that represent Melissa, her team and other teams within the organisation overlap, reflecting their being “interconnected” and working together. This contrasts with the “company itself,” represented by a rectangle “on the fringes” in the top right-hand corner. The contrasting shapes and composition of the map along the diagonal axis suggest that the organisation itself exists at a distance and disconnected from the individuals within it. Through this composition and use of shapes, Melissa counterpoints her sense of being closely connected to her coworkers with the disconnection and loneliness she feels in relation to the organisation.

Excerpt from Melissa’s description of her drawing: *“So, the square was supposed to be kind of representative of*,* like*,* the company. Um*,* and then the circle was meant to be, like, representative of all of the*,* kind of*,* the different teams*,* then like*,* the smaller circle was*,* like*,* our team*,* then the smaller circle below there was just like me as an individual*,* like a small part. But I put it all with, like*,* the teams [are] kind of interconnected*,* but not*,* like*,* fully in line with each other and they’re kind of on the edges of the company itself because the company*,* they don’t really understand the practicalities of what we do and*,* like*,* the day to day… So that’s why I kind of put it just on the fringes because we’re kind of like*,* there’s a disparity there*,* I think.”*

### Theme 3: disconnection from society

A number of participants described experiencing loneliness as disconnection from society because of their job. For these workers, loneliness centred on the perception that their job and, as a result, they themselves were not valued or respected by society at large.

The pandemic was an opportunity for many participants to reflect on their job, particularly those who worked in ‘essential’ non-healthcare roles such as transport workers and teachers. These workers recounted their jobs receiving increased public attention while simultaneously experiencing deteriorating working conditions during the pandemic. These participants attributed their worsening working conditions to disregard for them and their jobs by the government and media, and centred descriptions of loneliness on a yearning to feel supported by these institutions and society more broadly. This was evident in Jack’s account, in which he described his loneliness in relation to inadequate government support for teachers and the education system, rather than his school or colleagues:

Interviewer: Do you have any ideas for what your school could do to help staff feel a bit less lonely, more connected?

Jack: It’s the government. The reason why is the government. They have provided some support, but it was awful. I don’t think there’s much the school can do. They try their best, but there’s not much they can do, really. I blame the current pressure on the government. It makes you wonder if anyone out there actually cares about teachers– that’s isolating.

Jack, teacher, aged 30–39.

These workers perceived a lack of support from the government as symptomatic of broader attitudes towards their job, specifically that they and their work are not valued or appreciated. In this context, loneliness is experienced as a yearning to feel like a valued and accepted worker and member of society, over and above one’s satisfaction with their relationships with other individuals.

Participants also described feeling excluded from dominant narratives of working during the pandemic such that their experiences are not understood by the public or reflected in public memory. A number of essential workers pointed to government policies and media narratives that treated office workers as the norm, while their experiences were disregarded:

Interviewer: Do you think that people’s work can play into feelings of loneliness?

Sally: Yes, absolutely. I have felt lonely because of work during the pandemic, definitely.

Interviewer: That’s really interesting, you know, because earlier you mentioned that you enjoyed your relationships with your colleagues, being able to see them at work during lockdown. Can you tell me more about feeling lonely at work?

Sally: I didn’t feel supported. I don’t think anybody understood. You know, every time they think, you know, “train staff, all they do is complain about money.” Never. It’s about safety, really. It’s all safety. And just in terms of just wanting to be valued, I suppose. Everybody wants to be valued… They were all at home, furloughed, and we’re still working and you’re thinking, every day we were going in and keeping it up and nobody seemed to care.

Sally, transport worker, aged 50–59.

For Sally, her role in providing essential services was not valued and her experiences during the pandemic were not understood by society at large, which she experienced as lonely.

Importantly, this loneliness and sense of disconnection from society often contrasted with positive relationships with colleagues for these participants. In spite of the difficulties described above, a number of these participants described themselves as “lucky” or “privileged” to experience social interactions with their colleagues. As such, experiencing loneliness in relation to society was distinct from loneliness as dissatisfaction with workplace interactions and relationships.

## Discussion

This study offers a conceptual framework of workplace loneliness grounded in workers’ lived experiences that indicates that workplace loneliness comprises not only subjective dissatisfaction with interpersonal relationships at work but also a sense of disconnection from larger social groups and structures, particularly one’s employing organisation and society as a whole. These findings point to the need to expand existing conceptualisations to include organisational and societal context when attempting to understand, measure and address loneliness among workers.

The importance of disconnection from society and one’s organisation in workers’ experience of loneliness indicates that dominant definitions of workplace loneliness do not capture important aspects of the phenomenon. These findings are consistent with collective loneliness, in which individuals feel that they lack a valued social identity that allows them to connect to broader social groups such as their nation [17], as well as sociological and anthropological theorising that asserts that loneliness can be experienced in relation to “more than human” sources of connection [34] and locates individual experiences of loneliness as intertwined with broader social and economic systems [e.g. 16, 35]. Despite these developments, workplace loneliness is typically measured using short scales that only assess loneliness in the context of interpersonal relationships [7], leaving aspects of workplace loneliness identified in this study unmeasured and their impact on workers unknown. Adopting a broader concept of workplace loneliness is necessary to more fully understand the phenomenon and its impact on workers’ health and wellbeing.

This broader conceptualisation also points to possible risk factors for workplace loneliness that have not been investigated. While existing research has highlighted the role of organisational policies in precipitating workers’ loneliness [13], this has largely focused on how such policies influence the formation of interpersonal connections at work. Our results suggest that less obvious policies that affect whether workers feel valued by their employer, such as working conditions and leave entitlements may also play a role in workers’ loneliness, while individuals from marginalised groups may be vulnerable to loneliness because of barriers to obtaining decent work that is valued by their community [36, 37]. Similarly, negative attitudes towards one’s occupation may be a threat to workers’ sense of societal connection and precipitate loneliness such that workers in stigmatised or low prestige jobs may be at increased risk of loneliness.

Consideration of these contextual factors does not discount the role of interpersonal relationships in workplace loneliness. Our results also underline the importance of collegial relationships, with many participants centring connection with coworkers in their accounts of workplace loneliness. These findings build on research on loneliness in the general population which has widely considered the association between loneliness and relationships with a partner, relatives and friends [38], but has seldom considered professional relationships. Our findings demonstrate that routine interactions with colleagues can be a source of satisfying connection that could buffer the effect of unsatisfying personal relationships on loneliness and health, highlighting the need for greater consideration of professional relationships in loneliness research.

Our findings also suggest that yearning to feel that one’s authentic self is understood, valued and belongs are central features of how loneliness is experienced in relation to other individuals and social groups at work. This accords with existing understandings of generalised loneliness [34, 39], as well as accounts of loneliness from adolescents [40], young adults [41], clinical populations [42] and adults from across cultures [43] which conceptualise loneliness as an unfulfilled desire to feel valued, understood and a sense of belonging. As such, our findings suggest that work-related loneliness is experienced similarly to loneliness experienced in other populations or life domains (e.g. family, community). The importance of loneliness experienced in different domains is not well explored in adulthood and it is not clear whether satisfying relationships in one domain can compensate for loneliness in another.

Our findings also suggest that work-related loneliness is not only rooted in the workplace social environment, but also relates to professional identity, social narratives about different occupations and individuals’ perception of how they fit into society as a worker. Within this context, while work-related loneliness was experienced by workers most often within the work environment, it, at times, bled into workers’ personal time as a result of the pervasiveness of these narratives, identities and perceptions. The distinct and shared aspects of loneliness experienced at work and in other domains is further complicated by the complexity of social networks, in which relationships can transcend the boundaries of work, friendship, family and community. Future qualitative research exploring the intricacies of individuals’ social worlds could improve understandings of how loneliness operates within and across domains.

The key strength of this study is its focus on lived experiences of workplace loneliness among a diverse sample of UK workers. While this sample allowed us to capture a range of experiences from across the British working population, the focus on UK workers may limit the transferability of our findings to other locales. This study was conducted in a context in which loneliness is high on public agendas, and in which employment relations and occupational hierarchies have been shaped by particular social, political and historical factors which may impact how workers experience and understand loneliness. For example, workers in the UK have high employment protections and lower working hours relative to workers in low- and middle-income economies [44–46], for whom physical hazards and long working hours may take precedence over work-related loneliness. Similar research conducted in other contexts could deepen our understanding of workplace loneliness and illuminate the role of contextual factors in shaping these experiences.

Small numbers of participants from individual industries also limit our exploration of variation across occupations. Relationships may be particularly important for workers in less volitional or rewarding roles [47], while occupations involving close collaboration may be protected against colleague-related loneliness. As the sample covered a range of industries and some teamwork-intensive occupations in manufacturing and healthcare were not recruited, it was not possible to investigate group differences. Research examining specific occupations could elucidate how loneliness is experienced across occupational groups.

Conducting data collection during the COVID-19 pandemic may also limit the applicability of our findings outside periods of disruption. However, the impact of the pandemic on working patterns persists and remote working has become normalised in many industries, helping our findings to remain relevant to current working conditions. Further, for many participants the pandemic was an opportunity to reflect on their experience of different modes of working, facilitating discussion of their experiences of work and loneliness.

Our results highlight the need for greater consideration of societal and organisational connection in workplace loneliness research. Adapting more extensive instruments that capture collective dimensions of loneliness [17] or developing new measurement tools could improve our understanding of the impact of workplace loneliness on health and occupational functioning. Qualitative research interrogating the conceptual overlap of work-related loneliness and related constructs such as workplace belonging, dignity and mattering at work is also needed to understand their unique and shared roles in shaping workers’ health and wellbeing, and to connect the growing workplace loneliness literature [7] with developments in vocational and organisational psychology [48–51]. Our findings also underline the importance of collegial relationships and the work environment in experiences of loneliness, underlining the need to consider workplace relationships in loneliness research more broadly.

Our findings also underline the role of employers and organisations in ameliorating loneliness among workers. Employers and managers could work to prevent loneliness by fostering supportive collegial relationships through cooperative workplace cultures and supporting interactions among staff. Organisation-focused interventions that enable workers to feel supported and valued by their employer, as well as protections of labour standards, workers’ rights and access to decent and meaningful work [49] may also help in preventing loneliness. In light of the adverse health [1] and occupational consequences of loneliness [5, 6], these interventions may have economic benefits associated with greater work engagement and productivity, in addition to benefitting workers’ health and wellbeing.

## Electronic supplementary material

Below is the link to the electronic supplementary material.

Supplementary Material 1

## Data Availability

The dataset analysed is not publicly available due to lack of informed consent and ethical approval. Interested qualified researchers can contact the first author to propose collaboration and use of the data within the scope of participants’ consent and ethical approval.
