# Supplementary material for: Conceptualising workplace loneliness: a qualitative investigation with UK workers
Source: Soc Psychiatry Psychiatr Epidemiol. 2025 May 20;60(10):2325–37. doi: 10.1007/s00127-025-02925-0 (PMC12449417; doi:10.1007/s00127-025-02925-0)
Supplement: Supplementary file 1 — Supplementary Material 1 [file 127_2025_2925_MOESM1_ESM.docx]

**Conceptualising workplace loneliness:
A qualitative interview study with UK workers**

*Social Psychiatry & Psychiatric Epidemiology*

Bridget T. Bryan,^1^ Elena Triantafillopoulou,^1^ Vaughan Parsons,^2,3^Louise Arseneault,*^1^ and Timothy Matthews,*^4^

^1^ Social, Genetic and Developmental Psychiatry Centre, Institute of Psychiatry, Psychology and Neuroscience, King's College London, London, UK

^2^ Occupational Health Service, Guy’s and St Thomas NHS Foundation Trust, London, UK

^3^ School of Life Sciences and Medicine, King’s College London, London, UK

^4^ School of Human Sciences, University of Greenwich, London, UK

* Co-last authors

Correspondence concerning this article should be addressed to Professor Louise Arseneault, Social, Genetic and Developmental Psychiatry Centre, Institute of Psychiatry, Psychology and Neuroscience, 16 De Crespigny Park, London, United Kingdom SE5 8AB. Phone: +44 (0)20 7848 0873. Email: [louise.arseneault@kcl.ac.uk](mailto:louise.arseneault@kcl.ac.uk)

***Supplementary material***

[Supplement A. Social media advertising strategy 2](#_Toc195522676)

[Supplement B. Participant information sheet 5](#_Toc195522677)

[Supplement C. Pre-interview survey 9](#_Toc195522678)

[Supplement D. Semi-structured interview guide 14](#_Toc195522679)

[Supplement E. Reflexive practice 15](#_Toc195522680)

# Supplement A. Social media advertising strategy

Participants were recruited using a maximum variation sampling strategy, in which a diverse sample is recruited to allow for the analysis of a wide range of experiences of the phenomenon of interest (Guest et al. 2013). Considering the variety of experiences of work in the UK, we aimed to recruit a sample of workers diverse in gender, ethnicity and age, and from a range of industries and occupations. As social media recruitment strategies can increase access to populations hesitant or less likely to participate in research (Sanchez et al. 2020), participants were recruited using social media advertisements to facilitate the recruitment of a diverse sample and align with the online data collection method.

The study was advertised on the social media platforms Instagram and Facebook. Advertisements were designed to appeal to workers from a range of occupations and demographic groups, and with varying experiences of loneliness and social connection at work. Photos and videos of workers from different industries were combined with information about the study in all advertisements. In light of past research suggesting that positively framed images with a person in the foreground are most appealing to potential participants (Ryan et al. 2019), advertisement photographs and videos were portrait-style, generally featuring a person with a positive facial expression looking towards the camera. These images were paired with text captions that were drafted to be clear and concise, and consistent with research suggesting that potential participants prefer brief captions that indicate the themes of the study and is consistent with the accompanying images (Batterham 2014). Previous research also suggests that online advertisements with a problem focus (“loneliness”) recruit individuals with elevated psychological distress compared with advertisements with a positive focus (“connect”, “social lives”) (Choi et al. 2017), such that emphasising loneliness in advertising materials may risk oversampling individuals with significant, current experiences of work-related loneliness. As we aimed to recruit individuals with and without current experiences of workplace loneliness, a combination of problem-focused and positively-focused advertisements were used (Table S1). Initially, multiple versions of the advertisements were posted to Instagram and Facebook. The relative performance of each advertisement in recruiting participants from across demographic groups was monitored in Meta Ads Manager. Based on this information, advertisements were adjusted and refined to increase their effectiveness in recruiting a diverse sample of participants to the study.

Potential participants who clicked the advertisements were taken to the expression of interest (EoI) page on the study website (Figure S1). The EoI page contained an overview of the purpose of the study and what participation would involve, and a form to enter their first name and email address to express interest in participating. After submitting their details in the EoI form, prospective participants were contacted by email with the participant information sheet and a link to the online consent form and pre-interview survey to complete using the Qualtrics online survey platform (Qualtrics 2021).

Participants were offered a £20 gift voucher as a thank you for their participation. Incentives were provided to encourage recruitment (Kelly et al. 2017) and highlight to participants that their time, effort and insights were valued by the researchers (Wiles 2012). As the median hourly pay in the UK is approximately £15, the £20 token of thanks was proportionate to approximately 1.5 hours of participation without acting as an inducement that could impact with the voluntariness of participants’ consent.

**Table S1. Advertisement caption text**

Advertisement captions were drafted in two parts. Part A text was displayed first and was intended to draw viewers’ attention and highlight the key themes of the research. Part B text was then displayed, indicating what participation in the study involved and/or how potential participants can learn more about the study. Different combinations of A and B text were used to create different adverts.

| **PART A** | **PART B** |
| --- | --- |
| How has COVID impacted how you work and connect? | Join an online interview and receive a voucher as thanks |
| We’re researching loneliness and work during COVID | Join an online interview and receive a £20 voucher as thanks |
| We’re researching experiences of work and loneliness during the pandemic. | Join an online interview and contribute to our study of work, loneliness and health. |
| We’re researching/studying experiences of work during the pandemic | We want to hear from you! |
| We’re researching/studying the impact of COVID on work and loneliness | We want to hear from people like you. |
| We’re researching/studying the impact of COVID on workers | We want to hear your story. |
| Help us understand how you work and connect during COVID | Share your experience in a confidential online interview |
| The way we work has changed | Share your experience in an [online] interview |
| The pandemic has changed how we work and connect | Share your story in an [online] interview |
| Have you been working during the pandemic? | Share your story in an interview and receive a voucher as thanks |
| Worked during the pandemic? | Join an online interview and share your experience |
| COVID has changed how we work and connect |  |

**Figure S1. Recruitment website home page and expression of interest page**

| 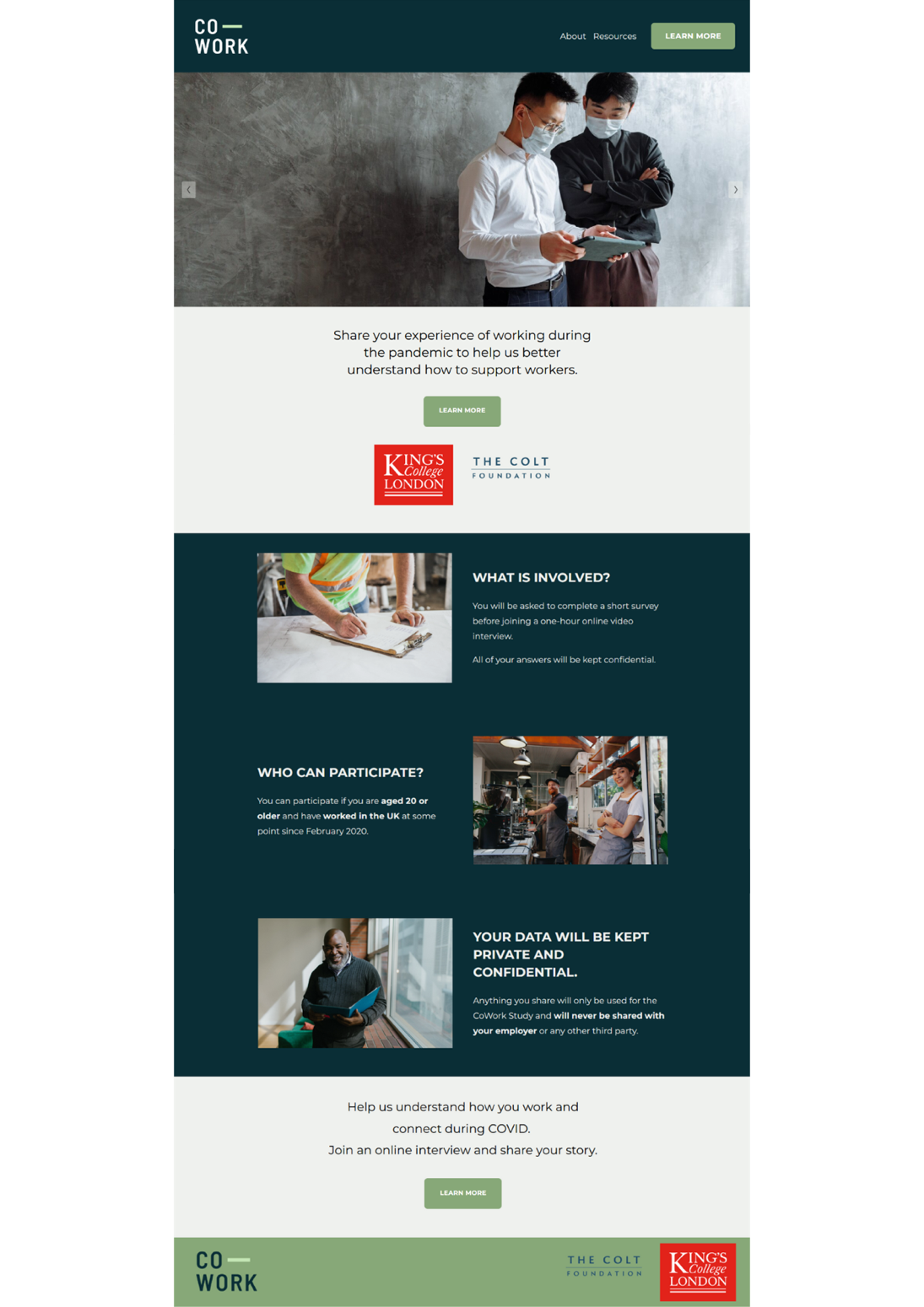A. Website homepage | 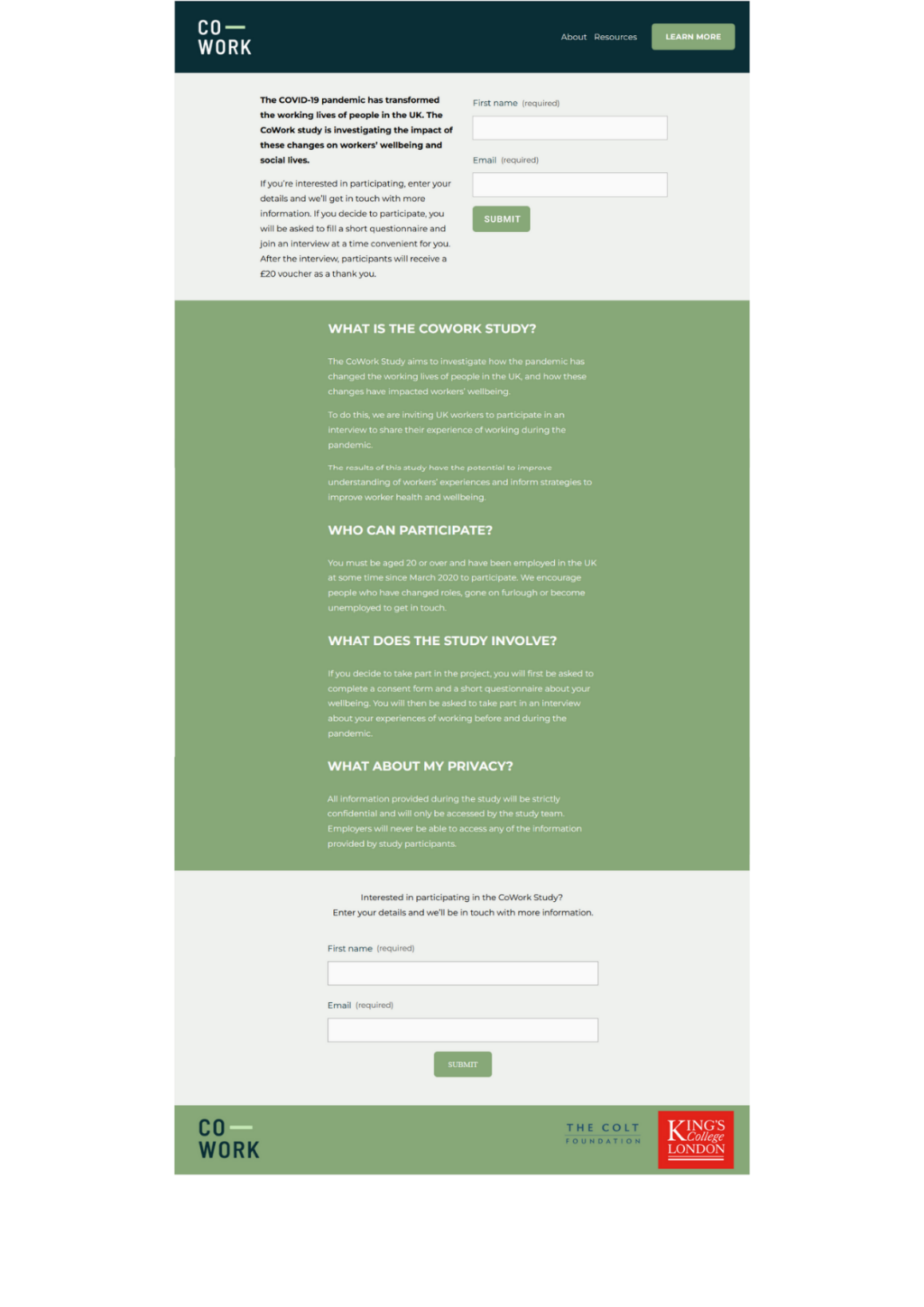B. Expression of interest page |
| --- | --- |

#

**References**

Batterham, PJ 2014, ‘Recruitment of mental health survey participants using Internet advertising: content, characteristics and cost effectiveness’, *International Journal of Methods in Psychiatric Research*, vol. 23, 184-191. <https://doi.org/10.1002/mpr.1421>

Choi, I, Milne, DN, Glozier, N, Peters, D, Harvey, SB, & Calvo, RA 2017, ‘Using different Facebook advertisements to recruit men for an online mental health study: Engagement and selection bias’, *Internet Interventions*, vol. 8, 27-34. <https://doi.org/10.1016/j.invent.2017.02.002>

Guest, G, Namey, EE & Mitchell, ML, 2013, *Collecting qualitative data: A field manual for applied research,* Sage, Los Angeles.

Sanchez, C, Grzenda, A, Varias, A, Widge, AS, Carpenter, LL, McDonald, WM, Nemeroff, CB, Kalin, NH, Martin, G, Tohen, M, Filioppou-Frye, M, Ramsey, D, Linos, E, Mangurian, C & Rodriguez, CI, 2020, ‘Social media recruitment for mental health research: a systematic review’, *Comprehensive Psychiatry*, vol. 103, 152197. <https://doi.org/10.1016/j.comppsych.2020.152197>

Ryan, J, Lopian, L, Le, B, Edney, S, Van Kessel, G, Plotnikoff, R, Vandelanotte, C, Olds, T & Maher, C 2019, ‘It’s not raining men: a mixed-methods study investigating methods of improving male recruitment to health behaviour research’, *BMC Public Health* vol. 19, 814. <https://doi.org/10.1186/s12889-019-7087-4>

Qualtrics, 2021, *Qualtrics*, accessed October 2021, Provo. [www.qualtrics.com](http://www.qualtrics.com)

Kelly B, Margolis M, McCormack L, LeBaron PA & Chowdhury D, 2017, ‘What affects people’s willingness to participate in qualitative research? An experimental comparison of five incentives’, *Field Methods*, vol. 29, no. 4, pp. 333-350. <https://doi.org/10.1177/1525822X17698958>

Wiles, R, 2012, *What are qualitative research ethics?* Bloomsbury, London.

# Supplement B. Participant information sheet

**
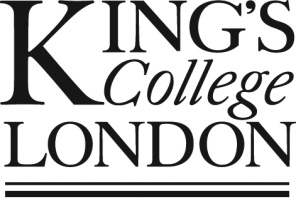
INFORMATION SHEET FOR PARTICIPANTS**

**YOU WILL BE GIVEN A COPY OF THIS INFORMATION SHEET**

*Ethical Clearance Reference Number:* HR/DP-20/21-22104

**Experiences of connecting, working and loneliness**

**during the COVID-19 pandemic (CoWork)**

We are a team of researchers studying experiences of working during the COVID-19 pandemic and would like to invite you to participate in this study. This study forms part of a PhD research project. Before you decide whether you want to take part, it is important for you to understand why the research is being done and what your participation will involve. Please take some time to read the following information carefully and discuss it with others if you wish. Let us know if there is anything that is not clear or if you would like more information. Contact details can be found at the bottom of this form.

**What is the purpose of the project?**

This project aims to investigate how the pandemic has changed the working lives of people in the UK, and how these changes have impacted workers’ wellbeing, health and feelings of connection and loneliness.

**Why have I been invited to take part?**

You are being invited to participate in this project because you are aged 21 or over and have worked in the UK at any point since the beginning of the first COVID-19 lockdown in March 2020.

**What will happen if I take part?**

If you choose to take part in the project you will first be asked to complete a consent form and short online questionnaire about your occupation and wellbeing. The questions will ask you about your life, such as your gender, ethnicity and the industry you work in. You will also be asked questions about your emotions and psychological wellbeing. You will not be asked to disclose the identity of your employer, and you can skip any questions you do not wish to answer.

You will then be asked to take part in an online interview about your experiences of working before and during the pandemic. The interview will take place on Zoom for approximately 60 to 90 minutes, and will be scheduled at a time which is convenient for you. Interview questions will explore how working has changed for you during the pandemic, and how this has affected how you socialise, as well as your mental health and wellbeing. You will not be asked to disclose the name or details of your employer during the interview. The interview’s audio and video will be recorded with your permission, so that we can transcribe the interview.

The interview will also include an exercise where you will be asked to create a map of your workplace relationships using an online drawing tool, and then discuss this with the interviewer.

**Do I have to take part?**

Participation is completely voluntary. You should only take part if you want to, and choosing not to take part will not disadvantage you in any way. Once you have read the information sheet, please contact Bridget at [cowork-study@kcl.ac.uk](mailto:cowork-study@kcl.ac.uk) if you have any questions that will help you decide whether to take part. If you decide to take part, we will ask you to complete a consent form and you will be given a copy of this consent form to keep.

**Incentives**

If you choose to participate in this study we will send you a code for a £20 Love-to-Shop e-voucher after the interview as a thank you for your time.

**What are the possible risks of taking part?**

We are inviting participants to talk about their experiences of work during the pandemic and its impact on their health and wellbeing. Any potential risks of participating would come as a result of disclosing a past or present distressing experience during the interview. Sometimes, people can find talking about the COVID-19 pandemic, their employment, health or wellbeing sensitive or uncomfortable. If this is true for you please mention it to the interviewer, and you are welcome to take a break at any time. Only talk about what you want to talk about. If you want to stop participating, you can do so at any time throughout the interview without giving a reason by speaking to the interviewer. All participants will receive information about some services that can provide some support.

**What are the possible benefits of taking part?**

If you choose to participate, you will help us understand how working and connecting has changed during the pandemic, and how this has impacted the health and wellbeing of workers.

Participants sometimes find it helpful to share their story with researchers, but it is important to know that research is not the same thing as counselling. We will provide all participants with a list of useful contacts, which can be used to seek appropriate help if needed.

You can also receive a copy of our final report or a lay summary describing the main findings from this study. This is optional and you do not have to receive any additional information regarding this study in the future if you do not want to.

You will also receive a £20 Love-to-Shop voucher after the interview as a thank you for your time.

**Data handling and confidentiality**

We take your privacy and confidentiality very seriously. Your data will be processed in accordance with the General Data Protection Regulation 2016 (GDPR). If you would like more information about how your data will be managed in accordance with GDPR please visit the link below: <https://www.kcl.ac.uk/research/support/research-ethics/kings-college-london-statement-on-use-of-personal-data-in-research>

All personal identifiable information about you is regarded as strictly confidential and it will be stored separately to the information you provide as part of the interview. All participants will remain anonymous and will be given an anonymous code number which will be used as an identifier throughout the research study. Only the research team directly involved in the study will be able to trace the information you have given to your personal details.

The interview will be audio and video recorded, and these recordings will be transcribed. The recording will be destroyed after it is transcribed. These transcriptions will be used with the drawings made during the interview for analysis. A code number will be attached to the audio recording, the interview transcript and your drawing to help protect your confidentiality. The audio recordings, transcripts and drawings will only be identifiable by the code number. Audio files, transcripts and drawing files will be password protected and stored electronically in a secure, encrypted folder on King’s College London’s SharePoint.

When we report the data, different names (pseudonyms) will be used to refer to all participants to help protect your confidentiality. We will also anonymise the names of any people, employers or organisations you talk about to help conceal your identity and maintain the confidentiality of the data you provide.

Information provided during the interview will be completely confidential. Confidentiality may be breached only when your life or someone else’s life is considered to be at risk, such as, in cases where suicidal thoughts or plans are disclosed. If this is to happen, our aim will be to make sure you get information on the help and support you may need.

The data you provide will only be accessed by the research team and will not be shared with any third parties. Your employer and government departments will not be able to access any of the information you provide while participating in the study.

Research data will be retained for 10 years after the completion of this research project according to King’s College London’s Records and Data Retention Schedule: <https://www.kcl.ac.uk/aboutkings/orgstructure/ps/audit/records/retention>

**What if I change my mind about taking part?**

You are free withdraw at any point of the project, without having to give a reason. Withdrawing from the project will not affect you in any way. You are able to withdraw your data up until 1 August 2022, when the analysis will be written up. If you choose to withdraw from the study, we will not retain any information that you have provided.

**How is the project being funded?**

This project is funded by a PhD fellowship from the [Colt Foundation](https://www.coltfoundation.org.uk/).

**What will happen to the results of the project?**

The results of the project will be summarised in a PhD thesis, scientific publications and conference/workshops. It will not be possible to identify you from any publications or presentation of the results.

**Who should I contact for further information?**

If you have any questions or require more information about this project, please contact us using the following contact details: *Bridget Bryan,* [*cowork-study@kcl.ac.uk*](mailto:cowork-study@kcl.ac.uk)*, Centre for Society & Mental Health, King’s College London, 16 De Crespigny Park, London SE5 8AF.*

**What if I have further questions, or if something goes wrong?**

If this project has harmed you in any way or if you wish to make a complaint about the conduct of the project you can contact King's College London using the details below for further advice and information: *Louise Arseneault,* [*louise.arseneault@kcl.ac.uk*](mailto:louise.arseneault@kcl.ac.uk)*, Centre for Society & Mental Health, 16 De Crespigny Park, London SE5 8AF.*

***Thank you for reading this information sheet and for considering taking part in this research.***

# Supplement C. Pre-interview survey

**Part 1: Demographic questions**

1. What is your age?

- 21 – 29 years
- 30 – 39 years
- 40 – 49 years
- 50 – 59 years
- 60 years +

1. What is your gender identity?

- Man
- Woman
- Non-binary / other gender
- Prefer not to say

1. What are your preferred pronouns?

- She/her
- He/him
- They/them
- Other (specify)

1. What is your ethnic group? If you identify as having mixed ethnicity/multiple ethnic groups, please choose all options that apply.^1^

White

- White British
- Any other White background (please describe)

Asian/Asian British

- Indian or Indian British
- Pakistani or Pakistani British
- Bangladeshi or Bangladeshi British
- East or South East Asian or ESEA British
- Any other Asian background (please describe)

Black/African/Caribbean/Black British

- Black African
- Black Caribbean
- Black British
- Any other Black/African/Caribbean background (please describe)

Other ethnic group

- Arab or Arab British
- Any other ethnic group (please describe)

1. Do you identify as a D/deaf or disabled person, or as having a long-term health condition?

- Yes
- No
- Prefer not to say

**Part 2: Work questions**

1. Which of the following best describes the industry you work in or worked in last? ^2^

- Agriculture, Forestry and Fishing
- Accommodation and Food Service Activities
- Administrative and Support Service Activities
- Arts, Entertainment and Recreation
- Construction
- Education
- Electricity, Gas, Steam and Air Conditioning Supply
- Financial and Insurance Activities
- Human Health and Social Work Activities
- Manufacturing
- Mining and Quarrying
- Media, publishing and communications
- Other Service Activities
- Professional, Scientific and Technical Activities
- Public Administration, Defence, and Social Security
- Real Estate Activities
- Tourism
- Transportation and Storage
- Water Supply, Sewerage, Waste Management and Remediation Activities
- Wholesale and Retail Trade (including Repair of Motor Vehicles and Motorcycles)
- Other (specify)

1. Please select which have applied to you since March 2020

- Worked at my usual workplace
- Worked from home
- Worked as an essential worker
- Was furloughed
- Lost my job or stopped working
- Started a new job
- Reduced work hours
- Was unable to work for more than two weeks because of sickness or disability

**Part 3: Loneliness (University of California Los Angeles [UCLA] three-item loneliness scale^3^ + 1 direct item)**

The next questions are about relationships with others. For each one, please say how often you feel that way.

How often do you feel…

1. You lack companionship?

- Never
- Hardly ever
- Some of the time
- Often

1. Left out?

- Never
- Hardly ever
- Some of the time
- Often

1. Isolated from others?

- Never
- Hardly ever
- Some of the time
- Often

1. How often do you feel lonely?

- Never
- Hardly ever
- Occasionally
- Some of the time
- Often or always

**Part 4: Psychological distress (General Health Questionnaire 12-item^4^)**

We would like to know how your health has been over the last couple of weeks. Have you recently…

1. Been able to concentrate on what you're doing?

- Better than usual
- Same as usual
- Less than usual
- Much less than usual

1. Lost much sleep over worry?

- Not at all
- No more than usual
- Rather more than usual
- Much more than usual

1. Felt you were playing a useful part in things?

- More so than usual
- Same as usual
- Less useful than usual
- Much less than usual

1. Felt capable of making decisions about things?

- More so than usual
- Same as usual
- Less so than usual
- Much less than usual

1. Felt constantly under strain?

- Not at all
- No more than usual
- Rather more than usual
- Much more than usual

1. Felt you couldn't overcome your difficulties?

- Not at all
- No more than usual
- Rather more than usual
- Much more than usual

1. Been able to enjoy your normal day-to-day activities?

- More so than usual
- Same as usual
- Less so than usual
- Much less than usual

1. Been able to face up to your problems?

- More so than usual
- Same as usual
- Less able than usual
- Much less than usual

1. Been feeling unhappy and depressed?

- Not at all
- No more than usual
- Rather more than usual
- Much more than usual

1. Been losing confidence in yourself?

- Not at all
- No more than usual
- Rather more than usual
- Much more than usual

1. Been thinking of yourself as a worthless person?

- Not at all
- No more than usual
- Rather more than usual
- Much more than usual

1. Been feeling reasonably happy, all things considered?

- More so than usual
- Same as usual
- Less so than usual
- Much less than usual

**Part 5: Interview planning and conclusion**

We look forward to scheduling an online interview at a time that is convenient for you. Please enter the days and times that you are generally available in the table below.

|  | Morning | Afternoon | Evening |
| --- | --- | --- | --- |
| Monday |  |  |  |
| Tuesday |  |  |  |
| Wednesday |  |  |  |
| Thursday |  |  |  |
| Friday |  |  |  |
| Saturday |  |  |  |
| Sunday |  |  |  |

Thank you for taking the time to complete this survey. We will be in touch by email in the coming days to arrange an interview.

**References**

^1^ Response options adapted from the UK Office for National Statistics (ONS) recommended groups for England. ONS (2022) *Measuring equality: A guide for the collection and classification of ethnic group, national identity and religion data in the UK.* [bit.ly/44K3Str](https://emckclac-my.sharepoint.com/personal/k1780970_kcl_ac_uk/Documents/2.%20PhD%20&%20studentship%20applications/3.%20Qualitative%20project/4.%20Data%20+%20analysis/bit.ly/44K3Str)

^2^ Response options drawn from the UK Standard Industrial Classification (SIC) used in the UK Census.
ONS (2009) *UK Standard Industrial Classification of Economic Activities (SIC 2007): Structure and Explanatory Notes*. Palgrave Macmillan. [bit.ly/3Pdh5F8](http://www.siccodesupport.co.uk/sic-division.php)

^3^ Hughes, M. E., Waite, L. J., Hawkley, L. C., & Cacioppo, J. T. (2004). A short scale for measuring loneliness in large surveys: Results from two population-based studies. Research on Aging, 26(6), 655–672. [doi.org/10.1177/0164027504268574](https://doi.org/10.1177/0164027504268574)

^4^ Goldberg, D., Gater, R., Sartorius, N., Ustun, T.B., Piccinelli, M., Gureje, O., & Rutter, C. (1997).  The validity of two versions of the GHQ in the WHO study of mental illness in general health care.  *Psychological Medicine*, 27, 191-197.

# Supplement D. Semi-structured interview guide

**Working history**

- You mentioned in the questionnaire that you work in the [industry]. Could you tell me a bit about your current/most recent job?
- Could you give me an idea of your experience of work so far? Maybe starting from your first job?
- Could you give me an idea of what you do at work day-to-day?

**Work during pandemic**

- Can you tell me a bit more about how your work has changed during the pandemic?
- What does a typical day look like for you? Was this different before the pandemic?
- How often would you say you chat with your colleagues?
- How do you stay in touch with your colleagues? If work from home: online meetings, email, chat, texting?
- People can range quite a lot in how much they like to socialise at work – where would you say you fall on that range?

**Social mapping task follow up questions**

- Can you explain your drawing to me?
- Where would you put yourself in the image?
- Is this how your relationships really are or how it should be?
- Is there something you would change?
- Has this changed since the pandemic? How/why has it changed?

**Loneliness at work**

- Loneliness can mean different things to different people. How would you define what loneliness is?
- Would you say you have felt lonely during the pandemic?

*If yes,* would you say your job has played a role in that? *Because of remote working/losing job/ furlough?*

- Do you think a person can feel lonely at work?
- Would you say that you have ever felt lonely at work?
- Do you think feeling lonely [at work] has impacted how you feel in yourself? In your health or wellbeing?
- Would you say that feeling lonely [at work] has impacted your work or how you do your work?
- What do you think employers can do to help their staff feel connected?

**Close:** is there anything that we haven’t talked about that you think is important?

# Supplement E. Reflexive practice

We engaged with reflexive practice throughout the project using two strategies. Firstly, the first author kept a reflexive research journal from the inception of the project. This journal was used to record and reflect her assumptions about work and loneliness, and how these shaped her decisions in planning and conducting the research, as well as her interpretation of the data. The second author also kept a reflexive journal while analysing a sample of the transcripts. Secondly, we used formal and informal discussions between all of the authors during the data analysis and write-up stages of the project as a space for reflection, aligning with a ‘kitchen table reflexivity’ approach (Kohl & McCutcheon 2013). Formal discussions focused on the first author’s analytic observations and the developing themes and included reflections on how these aligned with or diverged from our expectations. These discussions facilitated critical interrogation of our identities and perspectives and added depth to the analysis and interpretation. Throughout this process, we particularly focused on how our experience of working as researchers in an office environment shaped our assumptions about working life, as well as how our assumptions about the nature and experiences of loneliness and preferences for social connection at work may influence our interpretation of participants’ experiences and narratives.

**References**

Kohl, E & McCutcheon, P, 2015, ‘Kitchen table reflexivity: negotiating positionality through everyday talk. *Gender, Place & Culture*, vol. 22, no. 6, pp. 747–763. <https://doi.org/10.1080/0966369X.2014.958063>
